# Protocol for a mixed methods exploratory investigation into the role and contribution of the healthcare assistant in out-of-hours palliative care

**DOI:** 10.1186/s12912-021-00570-x

**Published:** 2021-04-08

**Authors:** Felicity Hasson, Sonja McIlfatrick, Sheila Payne, Paul Slater, Dori-Anne Finlay, Tracey McConnell, Anne Fee

**Affiliations:** 1 Institute of Nursing and Health Research, School of Nursing, Ulster University, Shore Road, Newtownabbey, BT37 0QB Northern Ireland; 2 International Observatory on End of Life Care, Lancaster University, Lancaster, LA1 4YG UK; 3 Institute of Nursing and Health Research, Ulster University, Shore Road Newtownabbey, Belfast, Co. Antrim BT37 0QB Northern Ireland; 4 Marie Curie Hospice Belfast, 1a Kensington Road, Belfast, BT5 6NF Northern Ireland

**Keywords:** Health care assistant, Out-of-hours care, After hours, Palliative care, Mixed methods, Hospice

## Abstract

**Background:**

Most people spend their last year of life at home, with many wishing to die there, but patients may need access to care after hours. Out-of-hours palliative care is delivered by multi-disciplinary teams including Health Care Assistants (HCA). However, little is known about the role, contribution and impact Health Care Assistants have on out-of-hours palliative care services. The aim of this study is to examine the Health Care Assistant role, contribution and impact on service delivery and patient care in out-of-hours community palliative care provided by hospice organisations.

**Methods and analysis:**

A mixed methods exploratory study consisting of four phases. Phase one involves a scoping review to systematically map and identify gaps in policy and literature on the HCA role in out-of-hours palliative care. In phase two, all United Kingdom hospices will be invited to participate in an online census to enable the development of a typology of out-of-hours services and the contribution of the Health Care Assistant. During phase three organisational case studies representing different service types will collect information from Health Care Assistants, patients, caregivers and service managers to gather qualitative and quantitative data about out-of-hours service provision and the Health Care Assistant role. Finally, phase four will synthesize and refine results through online focus groups.

**Ethics and dissemination:**

Ethical approval has been obtained for phase two through Ulster University Research Governance Filter Committee, Nursing and Health Research. Findings will be disseminated through practitioner and/or research journals, conferences, and social media.

**Supplementary Information:**

The online version contains supplementary material available at 10.1186/s12912-021-00570-x.

## Strengths and limitations of this study

- This exploratory study uses mixed methods to gather data from multiple perspectives.
- For each out-of-hours service typology, we will develop an in-depth organisational case study to create an understanding of the service, the professionals involved and the role the Health Care Assistant plays.
- Limitations include the single point in time data collection, with data only drawn based on United Kingdom out-of-hours community service models.
- A further limitation is the study is observational rather than interventional.

## Background

Most of the last year of a person’s life is spent at home, no matter where the patient eventually dies [1, 2]. Despite careful planning, unexpected deterioration may occur leading patients and caregivers to access out-of-hours care [3, 4]. In the United Kingdom (UK), out-of-hours (OOH) care refers to care provided between 18:30–08:00 on weekdays and throughout the weekends [5]. A recent exercise examining patient priorities demonstrated that OOH palliative care services were integral to the care of patients at end-of-life [6]. In fact, it has been estimated that 30% of palliative care patients have accessed OOH services in the last days of life [7]. However, research in this area has been minimal [8] despite reports of patient safety concerns relating to medication, access to timely care, clinical information and skills and confidence of the OOH team [5].

Multi-disciplinary teams comprising GPs, District Nurses, Specialist Palliative Care Nurses and Health Care Assistants (HCAs), all with variable training in end-of-life care, have a vital role to play in delivering OOH care. Even though HCAs (also known as nursing auxiliaries and support workers) are not regulated nor subject to any formal national training standards, they are key in providing home-based palliative care [9–11]. Evidence suggests HCAs play a critical role in the provision of chronic and end of life care [12–16] providing most of the direct care [15], and are often the first to recognise and alert professionals to patient changes [17, 18]. In the home it has been suggested that HCAs often act as a go-between with the caregiver and healthcare professional, providing support and guidance to caregivers and patients on what to expect, and a reassuring presence up to, and at the point of death [12, 19]. However, research indicates that many HCAs feel under-prepared to deliver chronic and palliative care [20, 21].

The contribution and impact of HCAs on hospice OOH service models remains largely un-reported in the literature, even though the roles of other OOH palliative team members have been previously examined [4, 22–24]. HCAs are rarely recognised as an integral palliative care team member [20, 25, 26], however, evidence suggests that HCAs are an extremely valuable workforce who make a vital and significant contribution to OOH palliative care [12, 14, 16]. Thus, a clearer understanding of the HCA role in OOH palliative care provision is crucial to potentially improve effectiveness of multi-disciplinary team working within OOH palliative care. Furthermore, developing insight to the range of OOH community-based service typologies being offered across the UK, and the role of the HCA in each service model may also help to identify strategies to enable patients to remain and die at home.

The current COVID-19 pandemic has resulted in greater pressures than ever before on health and social care practitioners. The pandemic has also resulted in unprecedented restrictions in research data collection within health and social care in order to limit transmission of the virus and also to prioritise resources. Consequently, researchers have had to adapt to ensure that data collection adheres to both methodological and ethical considerations. Therefore, the methods of data collection in the present study have been adapted to take into account current restrictions.

## Methods and analysis

Mixed methods design incorporating both qualitative and quantitative methods will be used as it provides a useful approach when studying real life, contextual situations [27]. Explanatory sequential design is described as *“starting by collecting and analysing quantitative data, and then collecting and analyzing qualitative data in a second phase; and finally link the phases by using the quantitative findings to form the qualitative research questions, sampling, and data collection”* [27] (p.71). Although an explanatory sequential design will be adopted as the primary design, there will also be elements of exploration in order to gain insight into any inconsistencies [28, 29].

As the aim is to provide an in-depth understanding of the HCA role in OOH palliative care, the qualitative data will take priority. A pragmatic paradigm will be applied, intentionally engaging both sets of paradigms and their assumptions, for the research problem under study [30]. Data will be integrated within phases using data transformation and data typology techniques [31]. Full integration will occur when data phases are completed using data matrix [32], following the thread [33]; and merging in a critical interpretation of the synthesis [27]. The study comprises four phases.

### Phase 1: scoping review

The first phase involves a scoping review of policy and related literature to examine and map the types of evidence and policy that inform practice in this area as well as identify gaps in knowledge [34]. There is no existing published synthesis on the HCA role in OOH palliative care that integrates empirical and grey literature to map the body of literature on this complex and heterogeneous topic [35].

In order to enhance rigour and transparency, the six-stage framework for scoping reviews developed by Arskey and O’Malley [36], and Levac et al. [37], will be adopted in order to identify relevant literature. The six stages are:

*Stage 1: Identifying the research question* - this was developed through consultation with the research team and key stakeholders, for example `What is the role, responsibilities and contribution of HCAs to palliative and OOH care?’. *Stage 2: Identifying relevant studies* - this will be achieved by searching several electronic databases (i.e. CINAHL, MEDLINE, EMBASE, PsycINFO), using pre-specified eligibility criteria and supplemented by grey literature searches (e.g., Web of Science Conference proceeding, Grey Literature report, Open Grey). Search terms and strategy will be developed with input from the research team, key stakeholders and an experienced research librarian. Terms will be searched as both keywords in the title and/ or abstract and subject headings (e.g. MeSH) as appropriate. English language and date limits of the past 10 years will be applied. *Stage 3: Study selection* – this will consist of two researchers independently reviewing (a) title and abstract and (b) full texts. In the case of conflicting eligibility, consensus will be reached through discussion with a third investigator. *Stage 4: Data collection –* this will comprise a generic data extraction template, which will be designed in accordance with the study aims to extract study characteristics. The extraction template will be reviewed by the research team and key stakeholders and pre-tested prior to implementation. *Stage 5: Data summary and synthesis of results* – this will entail a mapping of the key concepts underpinning the research area, detailing the main sources and types of evidence available. *Stage 6: Consultation* - this will be undertaken with a patient-partner who will be engaged throughout the study acting as a consultant to ensure the patient voice is captured. In addition, the search approach will be informed throughout by key stakeholders who are subject experts. The scoping study findings will be used to provide a platform for the design of the subsequent phases of the investigation.

### Phase 2: Quantitative: survey of UK hospices

The second phase involves a census of hospices across the UK using a cross sectional survey. This will enable the development of a typology of OOH services and examination of the HCA workforce characteristics, roles, employment contracts, organisational and regulatory guidelines for service provision within community-based OOH care. These findings will establish a national overview of the HCA workforce and their role in community-based OOH service provision and provide a sampling frame for the selection of ‘cases’ for phase three.

#### Population and sample size calculation

All Directors of Nursing or Community Nursing Services Managers, who have responsibility for hospice workforce staff and expertise in service delivery, based in adult hospices across the UK will be targeted. There were 260 services identified from the Hospice UK Service Directory (retrieved November 2017), and the sample will comprise one member from each service. A power calculation (95% confidence level, 5% confidence interval, 50% response accuracy, population *n* = 260) indicates a required sample size of *n* = 156 participants (accommodating an anticipated non-response rate for 40% [38].

#### Data collection

An online survey will be developed in line with best practice guidelines [39, 40]. This will be a self-administered questionnaire and will facilitate the collection of data in a relatively cost-effective manner within a short time frame.

#### Questionnaire design & pilot study

The survey will be informed by phase one findings and will include forced-choice and short answer (open) responses. The questionnaire will be divided into four main parts. The first will examine the OOH service typology delivered, models of practice, practice characteristics, accessibility and usage rates. The second section will profile the HCA workforce identifying the number, type, training, and role in the delivery of OOH care provided for patients receiving hospice care at home. The third part will explore hospice characteristics, size, status, location, and population. The final part will consider how OOH services and the HCA role has been impacted by COVID-19. Survey findings will be used to identify the variables of case study sites. The instrument will be pilot tested for clarity and face validity by a panel of experts in healthcare workforce, hospice and palliative care.

#### Survey process

Each adult hospice organisation will be initially contacted by telephone to identify and confirm the contact details of the most appropriate senior person to contact. An email invitation with a hyperlink to the online survey will then be sent to a senior person (usually Director of Nursing/ Community Nursing Services Manager) with an alternative hyperlink to opt out. Participants will be given a three-week period to complete and return the questionnaire online to the research team. To enhance response rates, a reminder email will be issued one week after initial distribution, and if necessary, a final reminder email one week later. The survey will be closed after four weeks. A process of implied consent will be used whereby a returned questionnaire will indicate consent for the data to be examined in the study.

#### Data analysis

Data collected from the questionnaire will be cleaned and entered to SPSS (v23). Descriptive statistics will be generated, and normality of distribution assessed. Content analysis will be undertaken of free text replies, to code those answers into a meaningful set of categories that may lend themselves to further quantitative statistical analysis. In addition, to enhance response rates reminder letters and the response rates from the online survey will be calculated according to the Checklist for Reporting Results of Internet E-Surveys (CHERRIES) [41]. Findings from the survey will be used to inform the sampling matrix for the selection of case studies in phase three and areas of interest for data collection.

### Phase 3: Qualitative: organisational case studies

As previously mentioned, phase two will enable an overview of the OOH service delivery and HCA role, and will aid in the identification of informants from all the palliative care services for the next round of data collection. The third phase will involve qualitative case studies. It is anticipated that this will result in a better understanding of the topic through an examination of the HCA role, contribution and impact of community-based OOH services, and the extent to which they enable people to remain at home.

#### Population and sample size calculation

A sampling framework will be used to purposively select up to six organizational case studies [42], of OOH services from phases one and two. Whilst the sampling criteria will be informed from the findings of phases one and two, the criteria for selection of cases has been further defined as follows: 1) Different configurations of Hospice OOH community palliative care services such as Rapid Response OOH Teams, Hospice at Home, Night Nursing Services; 2) The service provision has a multi-disciplinary background (HCAs must be involved in the delivery of care). Each type of model will be represented by at least one case study and OOH service will constitute a unit of analysis. Maximum variety sampling from phase two (survey) will be used to ensure the selected cases incorporate key variables of interest, for example, utilisation of HCA role, size of service and geographical location. Selecting cases in this manner will help to provide insight into the nature and context of different configurations of hospice OOH palliative care services, and together will represent the diverse experiences of key stakeholders.

#### Data collection

Based on the process model advocated by Yin [43], the approach will employ a mixed methods design to gain in-depth understanding of the HCA role and contribution to OOH palliative care across service models. Data collection methods will include interviews and documentary analysis. The primary method of data collection will be semi-structured interviews, conducted through a virtual platform (such as Skype or Zoom), each lasting between 30 and 60 min. As an alternative, telephone interviews will also be offered to suit participants’ circumstances. Interviews will be undertaken with patients, caregivers and staff associated with each OOH hospice community service model including HCAs, generalist, specialist and senior managers to provide a contextual picture of HCAs role within OOH services. Interviews with patients and caregivers will explore the contribution of the HCAs in patient care. Interviews with HCAs will explore their training, experience, role and contribution in OOH care delivery. Interviews with staff associated with OOH care will examine their perceptions of the contribution and impact of the HCA role in providing OOH support and the nature of working relationships. Senior managers will be asked to provide information about the overall service, the role of HCAs in providing support, training, and other factors influencing service delivery. Agendas for the interviews will be informed by the aims of the study and phase one and two analyses. All instruments will be piloted at a non-participating hospice. Capturing the experience of services and their organisational context will also be achieved through analysis of site-specific documents. Policy and materials relating to OOH community service models, the HCA role and policies and structures of working will be reviewed for each hospice. In addition, socio-demographic and epidemiological data on the localities in which the case study sites are situated will also be analysed to provide background contextual information for each case.

#### Recruitment and informed consent

Prior to data collection a volunteer principal liaison person [44], knowledgeable about the service, will be identified and given further in-depth information about the study. It is anticipated that this individual will act as a central point of contact for the research team and interview participants. Prior to interviews, this individual will be engaged in order to learn more about the service and its wider setting and to assist with recruitment for interviews. Following this, the research team will conduct individual interviews with participants, patients, caregivers, HCAs, other health care professionals (HCP) and senior managers, either via a virtual platform (Skype or Zoom) or telephone.

A purposive sample [45] of patients and caregivers (*n* = 2–4) who meet inclusion criteria will be selected from each hospice site. Patients who are willing to take part in the study will be asked to nominate a family caregiver. Separate interviews will be carried out with the patient and their nominated family caregiver to explore experience of the role and contribution of the HCA. These will be followed up with a purposive sample of HCA (*n* = 1–3) and key members of the OOH multi-disciplinary team (*n* = 3–5) such as the GP, District Nurse, Specialist Palliative Care Nurse and/or other members of multidisciplinary team as required (e.g., social work), and will be selected from each hospice site (see criteria below). Senior managers and OOH service coordinators/managers (*n* = 2–3) will also be interviewed from each site. The sample size for qualitative research cannot be specified precisely in advance and data collection will continue until data saturation occurs. It will be deemed that data saturation has been achieved when no new information has been uncovered [46]. All interviews will take place at a time that is convenient to the participants. Participants’ demographic information will also be collected using a brief questionnaire.

Inclusion criteria: case studies

1. Patients must be receiving community based palliative care, over 18 years old, physically and mentally capable of participation (judged by HCP) and willing to provide consent.
2. Caregivers must be over 18 years old, nominated by the patient, physically and mentally capable of participation and willing to provide consent.
3. HCAs must be employed in the delivery of OOH care to palliative care patients in the community, over 18 years old and willing to provide consent.
4. HCPs must be Generalist or Specialist Palliative Care HCPs and willing to provide consent.
5. Managers must be a senior manager within Hospice with a responsibility for delivery of OOH service and/or HCA management and willing to provide consent.

#### Data analysis

Each interview will be digitally audio recorded (with written consent), transcribed and subject to a framework approach to data analysis to enable comparison to be undertaken across and within cases [47]. In phase three, data will be `embedded’ within several sources (patients, caregivers, and healthcare professionals). Within-case analysis will ensure that a detailed understanding of each case study has been achieved. Cross-case analysis will be undertaken to identify cross-cutting themes common to all cases, and contextual issues to specific to individual cases [44]. Themes and patterns of the secondary data will be analysed and contextual issues will be highlighted.

### Phase 4: on-line focus groups

#### Goal

The final phase comprises on-line focus groups. These focus groups will be undertaken to facilitate reflection and discussion of the study’s findings, and will contribute to the development of strategic recommendations relating to the HCA role in OOH community palliative care provision.

#### Population and sample size calculation

A purposive sample of key stakeholders will be invited to take part (four focus groups comprising 5–8 participants per group). Participants will be initially recruited from key workers and stakeholders (*n* = 8–10) who took part in phase three (case studies) and identified as knowledgeable informants about HCA role and OOH community palliative care (i.e. HCAs, senior managers and other health care professionals). In addition, a purposive snowball sample of key stakeholders including, patients (with appropriate support to attend) and family caregiver representatives (*n* = 6–8), service managers (*n* = 3–4) and policy makers (*n* = 3–4) will also be invited.

#### Data collection

Focus group participants will be drawn from each UK jurisdiction (N. Ireland, Scotland, Wales, England), to ensure equal representation across the British Isles. It is anticipated that each focus group will last 1–1.5 h, on a date to be agreed with stakeholders. Focus groups will be conducted virtually, using videoconferencing (such as Skype or Zoom).

Given that face-to-face focus groups are ‘unique’ in their ability to produce insight to a phenomenon through participant interaction [48, 49], it could be argued that the virtual nature of the online environment may restrict the capture of nuanced group dynamics [50]. However, there is also some evidence to suggest that there are few differences in the quality of data generated from face-to-face and virtual environments [51, 52].

Over and above the risks associated with face-to-face focus groups such as lack of participation, or one person dominating, [48] there are also risks specifically associated with online focus groups. These include – participants lack of knowledge about the use of technology; unsuitable environments resulting in distractions or interruptions (from colleagues or family); privacy and confidentiality issues.

In the present study several measures will be used to mitigate against such issues. For example, for participants who are unsure of the online environment, training and a test call will be offered. Specific information included in the Participant Information Sheet, and Consent Form, such as the importance of securing a private space for the duration of the focus group, may help to appraise participants of the online process and expectations [53]. In addition, participants will be asked, if possible, to wear headphones in order to ensure confidentiality [52].

#### Data analysis

Quantitative data will be collected using questionnaires designed to formulate a picture of participants socio-demographic details, experience and attitude towards HCA in OOH palliative community care. Questions will contain a mixture of open and Likert scale questions. With permission, focus group discussions will be audio recorded, transcribed and subjected to thematic qualitative analysis [54] to determine the central themes. Issues that stakeholders perceive as important, and the development of strategic recommendations relating to the HCA role in OOH palliative care provision will be highlighted.

#### Patient and public involvement

A patient partner has been engaged throughout the study acting as a consultant to ensure the patient voice is captured.

## Ethics and dissemination

Ethical approval has been obtained for phase two through Ulster University Research Governance Filter Committee, Nursing and Health Research. Ethical approval for the remaining phases will be sought in a sequential manner in line with research governance and ethical requirements. All study participants will receive an information sheet detailing the study background, aim, implications and the ethical aspects. In phase two, participants will be made aware that submission of their questionnaire implies consent. In phase three and four full written informed consent will be required from all participants prior to participation. Ethical principles relating to confidentiality and data handling will be observed. Data from the questionnaires will be anonymous. When reporting the results all identifying features will be removed and interviewees will be identified by a unique code that only the research team can access. All data will be stored, maintained and disposed of according to current data protection legislation [55].

### Dissemination plan

A dissemination strategy informed by **E**vidence-based **M**odel for the **T**ransfer and Exchange of **Re**search **K**nowledge (EMTReK) in Palliative Care [56], has been developed. EMTReK provides guidance in knowledge transfer and exchange dissemination plans to multiple stakeholders using a range of dissemination tools. For this study the key stakeholders would include academics, palliative and end of life care providers, commissioning organisations, community nursing service providers, patients, caregivers and the public. A multi-facetted approach will be used in disseminating findings to different stakeholders throughout and at the end of the study.

### Written information

Throughout the study participating hospices in phase three (case studies) will be provided with regular updates to communicate to staff (via newsletters) and the wider community (via social media). The full account of the research findings and a plain English summary for public and patient engagement will be published online and will be available to our research participants. We also aim to publish in several peer reviewed and practice-based journals to reach broad audience coverage. The team will publish articles about the research aims and outcomes, designed for the public, including newsletters, research summaries written in plain English and social media feeds such as blogs.

### Oral presentations

Abstracts for oral presentations will be submitted to international and national research forums such as e the Hospice UK conference and the European Association of Palliative Care Congress. At a managerial level, presentations regarding the implications of the findings to health service organisations will be made to service managers responsible for delivering OOH community services and management of HCA workforce.

### Social media

We will use social media channels (YouTube, Twitter) via Ulster University and establish a dedicated project Twitter account to update on progress and disseminate the work in accessible and engaging format.

### Public

Dissemination of findings aimed at the public will be facilitated through links with organisations including Marie Curie, National Council of Palliative care and Dying Matters.

## Supplementary Information

**Additional file 1.**

## Data Availability

The datasets used and/or analysed during the current study will be available from the corresponding author on reasonable request.

## Abbreviations

HCA: Healthcare assistant
OOH: Out-of-hours
PC: Palliative Care
