# Supplementary material for: Protocol for a mixed methods exploratory investigation into the role and contribution of the healthcare assistant in out-of-hours palliative care
Source: BMC Nurs. 2021 Apr 8;20:55. doi: 10.1186/s12912-021-00570-x (PMC8026390; doi:10.1186/s12912-021-00570-x)
Supplement: Supplementary file 1 — Additional file 1. [file 12912_2021_570_MOESM1_ESM.docx]

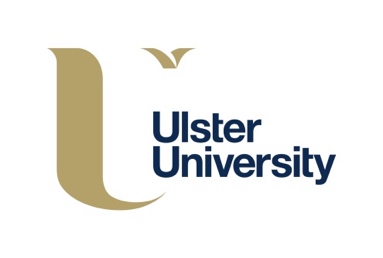


**NATIONAL SURVEY OF UK HOSPICE HEALTHCARE ASSISTANT WORKFORCE, AND THEIR ROLE IN OUT-OF-HOURS CARE PROVISION & IMPACT OF COVID-19**

***Thank-you for your interest in this survey, we greatly appreciate your time. The answers you provide will be reported anonymously and treated confidentially. Survey data will contribute to a better understanding of this important and under-researched area.***

***If you need to speak to anyone about any aspect of this survey, please contact Dr Anne Fee: a.fee@ulster.ac.uk***

**Instructions**

- Please read each question carefully and indicate your answer by ticking the box provided or writing in the space provided.
- The questionnaire consists of 4 sections. Completion of the survey should take around 15-20 minutes.
- Some survey questions require specific data that you may not have immediate access to. However, as this is an online survey, answers will be automatically saved to allow time to gather additional information.
- Once you have completed the questionnaire please submit it by clicking the ‘Submit’ box at the end of the survey.

***Completion of the questionnaire is voluntary and implies your consent to take part in this study.***

**Key concepts**

**Out-of-hours (OOH)** - In the UK, out-of-hours period is from 6.30pm to 8am on weekdays and all day at weekends and on bank holidays (NHS England 2017). However, variations in times exist.

**Healthcare Assistant (HCA) -** In the UK, Healthcare Assistants work in a range of clinical settings, they are not registered therefore work under guidance of a qualified healthcare professional. Healthcare Assistants are also known as nursing assistants, nursing auxiliaries, support workers or auxiliary nurses.

**Date of completion:**

**Job title of person completing the survey:**

**SECTION 1: PROFILE OF HOSPICE**

**1.1a Geography / demographics *(please tick/ record)***

Region hospice is in England  Scotland

Wales  Northern Ireland

b. Location of service: Town _____________________________________

County _____________________________________

c. Type of geographical area: Urban  Rural  Mixed

**1. 2a Hospice**

b. Is your hospice a Registered charity  Statutory (NHS)

Registered charity in Other

partnership with NHS

c. Which of these services are provided by your Hospice / Specialist Palliative Care Team? *(Select all that appl*y)

Inpatient  Day care  Out-patients  Home care  OOH care  Bereavement

Respite care

d. Currently (i.e. at today’s date), how many in-patient beds do you provide?

**____________________________________________**

e. During 2019, how many patients did you care for in the community?

_____________________________________________

**SECTION 2: OUT-OF-HOURS SERVICES**

2.1 Does your hospice provide out-of-hours care? Yes  No [*If No skip to Q4.1*]

2.1a What type of out-of-hours service(s) does your hospice provide? (Select all that apply)

Telephone advice for healthcare professionals only

Telephone advice for healthcare professionals, patients and caregivers

Rapid response

Care at home

2.1b Does your organisation have internal guidelines/policy regarding out-of-hours care? (i.e. what to do in an emergency). Yes  No

2.1c Does your organisation implement any national UK guidelines to determine how you deliver out-of-hours care? (i.e. Gold Standard Framework) Yes  No

***Model: Telephone Advice***

**Definition of telephone advice: Telephone advice is information/advice about any aspect of palliative care provided over the telephone (or by Skype), during all or some of the out-of-hours period.**

2.2a Does your hospice provide a dedicated telephone advice service?

Yes  No  (If ‘No’ please skip to Q2.3a)

2.2b Is this service available 24 hours per day, 7 days per week 365 days per year?

Yes  No

2.2c If ‘No’, Please indicate the times when your service is available. Select all that apply:

Evenings (for example 5pm-10pm)

Overnight (for example 8pm- 8am)

Weekends (Saturday and Sunday)

Bank holidays (including Christmas)

Other (please specify): ___________________________________________

2.2d What is the title of this service? __________________________

2.2e Is this service provided for: Healthcare professionals only

Patients and caregivers only

Mixture of healthcare professionals, patients & caregivers

2.2f Do eligibility criteria exist for this service? Yes  No

If yes, please specify:

______________________________________________________________________________________________________________________________________________________________________________________________________________________________________________________

2.2g What is the referral process for this service?

Professional

Self-referral

2.2h Which staff predominantly provide this service? (*Please tick a maximum of two options*):

Nurse(s)  Doctor  Healthcare Assistants  Other

(Please specify: _______________)

2.2i How is this service funded? ____________________________________________________________________________________________________________________________________________________________________

2.2j Please estimate how many calls this service receives annually:

0-50

51-100

101- 150

151-200

200+

***Model: Rapid Response***

**Rapid Response palliative care teams respond quickly to crises and emergencies.**

2.3a Does your hospice provide a Rapid Response service?

Yes  No  (If ‘No’ please skip to Q2.4a)

2.3b Is this service available 24 hours per day, 7 days per week 365 days per year?

Yes  No

2.3c If ‘No’, please indicate the times when your service is available. Select all that apply:

Evenings (for example 5pm-10pm)

Overnight (for example 8pm - 8am)

Weekends (Saturday and Sunday)

Bank holidays (including Christmas)

Other (please specify):___________________________________________

2.3d What is the title of this service? __________________________

2.3e Please indicate the tasks involved in this service. Select all that apply:

Pain management  Catheter care

Respiratory secretions  Personal care

Symptom control  Falls

Management of delirium Carer Support

/agitation  Crisis Response

Other (please specify): _______________________________________________________

2.3f Do eligibility criteria exist for this service? Yes  No

If yes, please specify

______________________________________________________________________________________________________________________________________________________________________________________________________________________________________________________

2.3g What is the referral process for this service?

Professional

Self-referral

2.3h Which staff predominantly provide this service? (*Please tick a maximum of two options):*

Nurse(s)  Doctor  Healthcare Assistants  Other

(Please specify: _______________)

2.3i How is this service funded? ____________________________________________________________________________________________________________________________________________________________________

2.3j Please estimate how many episodes of care are provided by this service annually:

0-50

51-100

101- 150

151-200

200+

***Model: Care at home***

**Terms used for this service: Palliative Homecare/community care/Hospice at Home/ palliative care support service/home nursing care/care at home/Virtual Ward.**

2.4a Does your hospice provide a care at home service?

Yes  No  (If ‘No’ please skip to Q3.1a)

2.4b Is this service available 24 hours per day, 7 days per week 365 days per year?

Yes  No

2.4c If ‘No’ lease indicate the times when your service is available. Select all that apply:

Evenings (for example 5pm-10pm)

Overnight (for example 8pm - 8am)

Weekends (Saturday and Sunday)

Bank holidays (including Christmas)

Other (please specify): ___________________________________________

2.4d What is the title of this service? _________________________

2.4e Which tasks are performed as part of this service? Select all that apply:

Pain management  Catheter care

Respiratory secretions  Wound care

Symptom control  Vital signs

Bed-bathing  Mobilising

Tube feeding  Mouth care

Bowel/ostomy care  Carer support

Domestic support  Personal care

Meal preparation  Falls

Other (please specify): ­­­­­­­­­­­­­­­­­­­­­­­_______________________________________________

2.4f Do eligibility criteria exist for this service? Yes  No

If yes, please specify:

________________________________________________________________________________

2.4g What is the referral process for this service?

Professional

Self-referral

2.4h Which staff predominantly provide this service? (*Please tick a maximum of two options):*

Nurse(s)  Doctor  Healthcare Assistants  Other

(Please specify: _______________)

2.4i How is this service funded? ____________________________________________________________________________________________________________________________________________________________________

2.4j Please estimate how many episodes of care are provided by this service annually:

0-50

51-100

101- 150

151-200

200+

**SECTION 3: IMPACT OF COVID**

- 1. Have you had any patients with confirmed (by test) or suspected (untested but clinical symptoms) of COVID-19 in your out-of-hours service?

Yes  No

- 1. Have you had any out-of-hours staff with suspected or confirmed COVID19?

Yes  No

3.3a Have your out-of-hours services changed in response to COVID-19?

Yes  No

3.3b If yes, in what ways (if any) have you changed how you are delivering OOH care

_________________________________________________________________________________________________________________________________________________________________________________________________________________________________

- 1. How would you say you are utilizing healthcare assistant staff compared to the same period last year?

A lot more

Slightly more

About the same

Slightly less

Much less

- 1. Have you changed how you deploy healthcare assistant staff?

Yes  No

If yes, please explain the changes and why you have made them.

______________________________________________________________________________________________________________________________________________________

- 1. Since COVID, how well would you say your out-of-hours service is integrated with other NHS primary or secondary care services?

No integration at all

Slightly integrated

Somewhat integrated

Very integrated

Extremely integrated

Any comments on integration:

_________________________________________________________________________________________________________________________________________________________________________________________________________________________________

3.7a Has COVID-19 changed the way you are supporting patients requiring out-of-hours care?

Yes  No

3.7b If yes, in what ways (if any) have you changed the way you are supporting patients requiring out-of-hours care?

_________________________________________________________________________________________________________________________________________________________________________________________________________________________________

3.8a Has COVID-19 changed the way you are supporting family members/those important to the patient out-of-hours?

Yes  No

3.8b If yes, in what ways (if any) have you changed the way you are supporting family members/those important to the patient out-of-hours?

_________________________________________________________________________________________________________________________________________________________________________________________________________________________________

- 1. What would you say are the three main challenges in delivering OOH care during the COVID-19 pandemic?

1.__________________________________________________________________________2.__________________________________________________________________________

3.__________________________________________________________________________

- 1. What would you say are the three main facilitators in delivering OOH care during the COVID -19 pandemic?

1.__________________________________________________________________________2.__________________________________________________________________________ 3.__________________________________________________________________________

- 1. Is there anything else you want to tell is about how your out-of-hours service has operated since COVID?

______________________________________________________________________________________________________________________________________________________

**SECTION 4: HEALTHCARE ASSISTANT WORKFORCE**

4.1a Currently, how many healthcare assistants (whole time equivalent) does your hospice employ within the:

In patient service  Community service

Out-of-hours services

4.1b How many are on each banding/pay scale?

Band 2  Band 3

Band 4  Other (*please explain*)

______________________

4.1c What training do they receive?

Mandatory  General Palliative care

Specialist palliative care  Bereavement

Other

4.1d What are the key tasks the healthcare assistant performs in out-of-hours care?

____________________________________________________________________________________________________________________________________________________________________

__________________________________________________________________________________

4.1e We would value any additional comments you may have. Please write comments below about out-of-hours services and the role of healthcare assistants within your hospice.

____________________________________________________________________________________________________________________________________________________________________

____________________________________________________________________________________________________________________________________________________________________

____________________________________________________________________________________________________________________________________________________________________

____________________________________________________________________________________________________________________________________________________________________

**THANK YOU FOR COMPLETING THIS QUESTIONNAIRE**

**PLEASE SUBMIT**

**SAVE & RETURN TO LATER**

For further details about any aspect of this survey, please contact:

Dr Anne Fee: a.fee@ulster.ac.uk or Dr Felicity Hasson: [f.hasson@ulster.ac.uk](mailto:f.hasson@ulster.ac.uk)
